# Assessment of the design and implementation challenges of the National Health Insurance Scheme in Nigeria: a qualitative study among sub-national level actors, healthcare and insurance providers

**DOI:** 10.1186/s12889-020-10133-5

**Published:** 2021-01-11

**Authors:** Gbadegesin O. Alawode, David A. Adewole

**Affiliations:** Department of Health Policy and Management, Faculty of Public Health, College of Medicine, University of Ibadan, Ibadan, Nigeria

**Keywords:** National Health Insurance Scheme, Stakeholders, Healthcare financing, Healthcare providers, Health maintenance Organisations, Universal health coverage, Nigeria

## Abstract

**Background:**

Health insurance is an important mechanism to prevent financial hardship in the process of accessing health care. Since the launch of Nigeria’s National Health Insurance Scheme (NHIS) in 2005, only 5% of Nigerians have health insurance and 70% still finance their healthcare through Out-Of-Pocket (OOP) expenditure. Understanding the contextualized perspectives of stakeholders involved in NHIS is critical to advancing and implementing necessary reforms for expanding health insurance coverage at national and sub-national levels in Nigeria. This study explored the perspectives of sub-national level actors/stakeholders on the design and implementation challenges of Nigeria’s NHIS.

**Methods:**

A descriptive case study design was used in this research. Data were collected in Ibadan, Oyo State in 2016 from health insurance regulators, healthcare providers, and policymakers. Key informant interviews (KII) were conducted among purposively selected stakeholders to examine their perspectives on the design and implementation challenges of Nigeria’s National Health Insurance Scheme. Data were analysed using inductive and deductive thematic approaches with the aid of NVIVO software package version 11.

**Results:**

Implementation challenges identified include abject poverty, low level of awareness, low interest (in the scheme), superstitious beliefs, inefficient mode of payment, drug stock-out, weak administrative and supervisory capacity. The scheme is believed to have provided more coverage for the formal sector, its voluntary nature and lack of legal framework at the subnational levels were seen as the overarching policy challenge. Only NHIS staff currently make required financial co-contribution into the scheme, as all other federal employees are been paid for by the (federal) government.

**Conclusions:**

Sub-national governments should create legal frameworks establishing compulsory health insurance schemes at the subnational levels. Effective and efficient platforms to get the informal sector enrolled in the scheme is desirable. CBHI schemes and the currently approved state supported health insurance programmes may provide a more acceptable platform than NHIS especially among the rural informal sector. These other two should be promoted. Awareness and education should also be raised to enlighten citizens. Stakeholders need to address these gaps as well as poverty.

**Supplementary Information:**

The online version contains supplementary material available at 10.1186/s12889-020-10133-5.

## Background

In improving access to quality healthcare services, the World Health Assembly in 2005 has increasingly called for countries to prioritise universal health coverage (UHC). This remains a viable means of providing appropriate promotive, preventive, curative, and rehabilitative services at an affordable cost for all [1]. Thus, globally, stakeholders have laid much emphasis on funding mechanisms of health systems [1]. Aside the tax-based (Beveridge model) method of health financing, the social health insurance (SHI) (Bismark model) which has its root in Germany in the nineteenth century is one of many approaches used to address the challenges related to providing access to health care services for the poor segments of the population [2]. However, other different models of health financing exist such as the Medical Savings Account – (self – reliant/funding) in Singapore [3] and the Affordable Care Act (ObamaCare) USA, Community Based Insurance, and Private Health Insurance. Healthcare financing plays a critical role in the strengthening of a nation’s health system which necessitates the implementation of sustainable health financing structures and monitoring of progress towards achieving UHC [4].

A health insurance scheme has been defined as an arrangement in which contributions are made by or on behalf of individuals or groups (members) to purchasing institution (a fund) which is responsible for purchasing covered services from providers on behalf of the members of the scheme, [5]. A social health insurance scheme involves contributions based on means and utilization based on need. It holds strong potential to improve financial protection and enhance utilisation among enrolled populations. This underscores the importance of health insurance as an alternative health financing mechanism capable of mitigating the detrimental effects of user fees, and as a promising means for achieving universal healthcare coverage [6].

The aim is to reduce out of pocket payment in all forms as this payment method reduces equity of access to health care especially among the poor [7, 8].

In Sub-Saharan Africa (SSA), the challenge of UHC is critical most especially in ensuring financial protection and access to needed health care for those outside the formal sector. This is due to constrained tax revenue in many countries, equity, and efficiency problems associated with contributory schemes for this group (Informal sector) [9]. The burden of health expenditures is mostly attributed to common endemic diseases; they constitute a majority of the public health problems because of their recurrent nature and are the major causes of morbidity and mortality [10]. The high level of OOP spending and paucity of insurance mechanisms to pool and manage risk form a major challenge to health care financing in Nigeria [11]. Poor financial capacity of consumers to pay for needed health services results in inequitable access to health care [12]. This has limited many Nigerians from accessing the needed healthcare services resulting in loss of productivity, poverty, poor health outcomes, and preventable deaths.

### The Nigeria National Health Insurance Scheme

The history of NHIS could be traced back to 1962. However, the scheme became operational in 2005 as a tripartite public-private arrangement among three main stakeholder operators; the NHIS, the HMOs and health care providers. The other stakeholder are the enrolees under the scheme [13].

The primary aim is to ensure UHC that could enable improved access to health services and thus, a better population health outcome. It had the goal to achieve UHC within a period of 10 years from its inception (2005–2015). While the NHIS shapes the health insurance policy by accrediting the HMOs that operate within the health insurance space, it also accredits health care facilities to provide the benefit packages to registered enrollees. The HMOs are in charge of purchasing health care services on behalf of the Scheme for registered enrollees.

The scheme has different programmes for different population groups in the country such as the formal and informal Sector Social Health Insurance Programme [14, 15]. NHIS is a pro-poor policy with the potential to promote access to needed quality health care among Nigerian populace and reduce the rate of uninsured as was reported in the ACA in America [16]. However, opinion is polarized among stakeholders on the efficacy of the scheme in addressing the health situation and poor health outcomes in the country [17].

Thus, there is a growing need to correct the persistent poor coverage by assessing the design and implementation challenges of the scheme. This will provide an objective assessment of the situation for policy actors.

## Methods

### Study design, population

The study was a descriptive case study design that employed qualitative methods using key informant interview (KII) with expert actors in the health insurance space in Oyo State, Nigeria. Nine KII were carried out among purposively selected health insurance stakeholders, consisting of 8 males and one female between the ages of 30 and 60 years, a mean age of 43.9 years who are major stakeholders (state political leader, heads of health insurance agency, managers of health maintenance organisations, heads of healthcare providers) whose organisation had been operating in the health insurance industry and providing services to enrollees for more than 6 months.

All the HMOs and almost all of the accredited healthcare service providers were located in Ibadan, the Oyo State capital. The three selected HMOs and three Health Care Providers (HCPs) were the most patronised and have the largest enrollee base. The Zonal and state coordinating offices of the NHIS were also located in Ibadan.

### Data collection

The key informant interview guide (see Additional file 1) was used to obtain the perspectives of nine identified stakeholders between August and October, 2016. These stakeholders and their organizations were involved in the design and implementation of the NHIS and they had the highest enrollee base. Visits and phone calls were made to book appointments and fix dates for each interview. Based on scheduled appointments, stakeholders were interviewed by the author (GOA), a Masters of Public Health student at the Department of Health Policy and Management, University of Ibadan, Nigeria. Interviews were tape-recorded with permission and informed consent obtained from stakeholders, and also, side notes were taken. The average length of the interview was 45 min. The interviews were conducted in English Language as the official language of communication. Stakeholders’ interviews ceased once saturation was reached while emerging themes were probed further. Interview guides were developed by author (GOA) with a guide from literature and with assistance from the supervisor (DA) and were tested for flow and coherence. Stakeholder interviews focused on the design and implementation mechanism of the scheme, implementation challenges, suggestions to solve identified challenges, awareness, and opinions on reforms and suggestions on how reforms at the subnational level could be implemented to expand coverage.

### Data management and analysis method

Data analysis was done using a mixed method of inductive and deductive thematic approach with the aid of N-VIVO software package version 11. Audio-taped interviews were transcribed verbatim, author GOA and an independent coder got familiarized with the data by reading through it many times during which initial codes were generated.

The generated themes were reviewed first at the level of coded data, then with the entire data set. Key themes were identified, while coding of several transcripts were done by two people (the lead author and an independent coder), independently to develop a thematic framework. Where there were disagreements between the two analysts, a consensus was reached amicably. Emerging themes were documented and analysed accordingly. Themes and narratives were interpreted within the context of the study. Themes were thereafter defined, named, after which the results were organised and written by the interview guides main domains: design and implementation mechanism of the scheme, implementation challenges, suggestions to solve identified challenges, awareness and views on reforms and suggestions on how reforms at the subnational level could be implemented.

### Trustworthiness

#### The person of the researcher

The researcher is a master’s student of public health with specialisation in public health policy, financing, and management. He has not worked in any organization and had no role outside of the Department of Health Policy and Management. Hence, there was no opportunity for him to influence the respondents’ responses.

#### Researcher’s roles in the study

As a master’s student, author GOA owns the study idea and developed it together with author DA. He took the lead in contacting necessary stakeholders such as the NHIS, HMOs and the HCPs to explain the purpose of the study, obtained permission to collect data.

#### Trustworthiness of the data

Interviews were conducted by Author GOA who was a master’s student of public health with experience in qualitative data collection. He also has requisite skills in communication, attention to detail, critical thinking, and ability to maintain quality. With the research team, he led the planning and scheduling of appointments with study participants, interviewing techniques and data collection and transcription, challenges, and how to overcome them. Before this, he has been trained on basic principles of research ethics with emphasis on confidentiality of shared information, benevolence, benefits, and risks among others.

To meet the credibility criteria, the guides were piloted for clarity and flow with members of the research team. The field pretest of the data collection instruments was carried out with representatives from the stakeholders who were not included in the study sample. Questions and comments were entertained, and useful amends were made to the data collection tools as appropriate. Also, data triangulation was applied which included several stakeholders with different institutional experiences and professional backgrounds as study participants. Two investigators collected and analysed the data using transcribed interviews alongside field notes and voice recordings. For transferability of the findings to different settings, we provided the sampling, sample size, interview procedure, findings and inclusion and exclusion criteria.

#### Ethical approval

The University of Ibadan/University College Hospital Ethical Review Committee approved this study (UI/EC/16/0234). A letter of introduction was written to all the stakeholders to be interviewed after which permission was granted for the purpose. Written informed consent was also obtained from every participant of the study.

## Results

Nine stakeholders were interviewed, two health insurance regulators, three healthcare providers, three health maintenance organisations and one state political leader. The composition is shown in Table 1.

**Table 1:** Types of Respondents Interviewed

Respondent	Total Number
State Political Leader	1
State Health Insurance Agency	1
NHIS	1
HCP	3
HMO	3
Total	9

### Design and implementation mechanism of the National Health Insurance Scheme in Nigeria

#### Funding mechanism

Generally, Health Insurance and Healthcare Providers stakeholders reported no federal government worker/employee is co-contributing into the scheme, however, a representative from the regulators- National Health Insurance Scheme (NHIS) further revealed that NHIS staff were already paying into the scheme. The finding is corroborated by quotes from key informant interviewees as stated below:

*“*I *have told you initially that all federal government staffs are on meritorium. I don’t know when the government will start deducting but the government will have a targeted day when they will start charging individual enrollees” (Healthcare Provider, code 002).*

*“No Nigerian enrollee except the staff of NHIS are paying for now. Every federal worker/employee in Nigeria are not paying dime except NHIS staffs. Federal government still pays on behalf of its workers.” (National Health Insurance Scheme).*

Stakeholders involved in the design of the subnational scheme (State Supported Health Insurance Scheme- SSHIS) remarked on the payment plan for the scheme when it becomes fully operational such as through the enrollees’ prepayment plan, government, international donors, proceeds of investment from the agency and the National Health Act.

*“Basically, money comes from enrollees. Enrollee’s prepayment plan. In many states, they do cross-subsidy or they do state subsidy …*. *Also, some international organizations that want to support state, proceeds of investment from the agency, National Health Act (State Health Insurance Agency).*

#### Category and utilisation of funds

All the stakeholders reported two major categories of funds such as capitation and fee-for-service used to purchase health services either at the primary, secondary or tertiary level for the enrollees depending on the total number of lives registered with both insurance providers and healthcare providers and referrals made. Aside from these fees, administrative fee is paid to the health maintenance organisations for the operational running of their services. Also, enrollees only pay 10% of the cost of drugs given by the providers. The quotes below highlight these views.

*“Capitation is 750 naira and that is for primary health care services. If there is any need for secondary, that is referral, the scheme will pay what we call fee-for-service. This is based on the total of number of the enrollees that registered with the facility per time irrespective of whether they access services or not” (National Health Insurance Scheme).*

*“750 for capitation, fee-for-service I think that should be like 90 naira around that figure, administrative charges 100 and something naira per enrollee” (Health Maintenance Organisation, code 004).*

However, for healthcare providers, stakeholders reported that the capitation is inclusive of their administrative fees. The quote below shows this.

*“750 naira for individual and you now multiply it by the number of enrollees you have including administrative charges. It also covers drugs but they will ask that patient to pay 10% on the cost of drugs” (Healthcare Provider, code 002).*

Stakeholders involved in the design of the State Supported Health Insurance Scheme (SSHIS) reported 600 naira per month and 7200 naira per annum as the basic standard subscription for enrollees into the scheme. The quote below highlights their opinion.

*“7,200 naira per annum. That’s for standard. We have golden and others. It’s in 3 stages. It is just 600 naira per month, which is fair enough” (State political leader).*

#### Sufficiency of capitation

Stakeholders reported varying opinions as to the adequacy of the capitation especially in line with current economic realities in Nigeria. Many of the respondents believed the capitation is adequate for the provision of healthcare based on the fact health insurance is a game of numbers and not all enrollees will access healthcare within a month or even throughout the year. While some reported that the capitation is not adequate and should be reviewed if the scheme is to be sustained.

*“As at the time this capitation was made they didn’t have this economic issues but even at that, it is still adequate because it is a game of numbers you see the game of numbers is a game of risks at the primary health care level the hospitals are the ones that bear the risk what is the risk they are bearing?...... At the secondary care level is the HMO, they give him about 90 naira per enrollee it is the HMO that bears the risk in a month. You may not have a single referral but what happens to the 90 naira, your gain. In a month you may have so many referrals so the HMO bears the risks and at the secondary health care level. So it is sufficient” (Health Maintenance Organisation, code 004).*

*“It is not adequate, peradventure, if NHIS wants to sustain this scheme they have to start the issue of co-payment is implemented as soon as possible” (Healthcare Provider, 003).*

According to stakeholders designing the SSHIS, the 600 naira per month capitation fee is not enough, the reason why the government and international organisations should contribute their quota:

*“Because the 600 is not enough, that is the reason why the government needs to contribute its own quota and federal government, state government, even international organization need to contribute their own quota as well” (State political leader).*

##### Reimbursement of HMOs and HCPs

Confirming the process of reimbursing health maintenance organisations and healthcare providers on the scheme, stakeholders remarked that government/NHIS pays capitation to the HMOs based on the number of lives under them, while the HMOs now pay HCPs based on the number of lives registered under them. However, fee-for-service is paid by HMOs to HCPs upon receipt of the bills. The below quotes show the views of stakeholders.

*“Government gives the HMO money based on the number of lives that they have and based on that they now pay the HCPs based on the number of lives they have.” (Health Maintenance Organisation, code 005).*

*“We pay monthly for their capitation but for their fee for service that is they send us their bills. Health … The total number of enrollees that register with them whether they come or not “(Health Maintenance Organisation, code 004).*

For the State Supported Health Insurance Scheme, stakeholders reported that HCPs will be paid 3 months upfront for the number of enrollees while HMOs will be paid required administrative charges when used:

*“We front-load capitation for them for 3 months. That’s what is called insurance. We front load the enrollees that are there to them whether they access services or not … .since HMO is not on a particular health plan there is no reimbursement. Anywhere we use them, we pay an administrative charge, that’s what they collect, standard. “(State Health Insurance Agency).*

### Assigning enrollees to HMOs and HCPs

Stakeholders reported that initially federal government enrollees/ministries were assigned to already existing HMOs who were duly registered with NHIS. But the approach has changed now as each HMO will have to market their services before getting new enrollees.

*“When NHIS started there were some HMO’s that were already in existence … when NHIS kick-started they noticed that which HMOs are going to help us run this system …*. S*o then people went to start canvassing for the ministries, they literally almost distributed the ministries to the existing HMOs. When eventually a letter has been written to you that congratulations you are officially the HMO for Nigeria police south-west what then happens, the HMO and its team applies for a registration form from NHIS” (Health Maintenance Organisation, code 004).*

“*… Then but, I don’t know because they are talking now that you have to market for your parastatal now. It is what you do now that will sell you to the people” (Health Maintenance Organisation, code 005).*

However, the scheme allows enrollees the freedom to choose or change any of the healthcare providers of their choice based on the satisfaction of services received. This finding is buttressed by the quote below:

*“… Enrollees have the freedom to choose wherever they want to. We don’t force any enrollee to choose any provider. However, if they have chosen and not satisfied with care they received … They have the right to change, after 6 months they can change their provider“(National Health Insurance Scheme).*

The majority of the stakeholders reported that enrollees’ choice of providers won’t necessarily lead to lopsidedness in the distribution of enrollees under the scheme, but will aid the scheme to be better. However, it was acknowledged that the quality of services and satisfaction received by the enrollees have a greater influence on their choice of providers. These views are supported by the quotes below:

*“No, you see it’s a matter of choice. You are at liberty to pick the hospital of your choice, so there is no way there won’t be lopsidedness, variations, there is going to be. Because if I am not getting the best service from your hospital, I will leave. I have told you initially is a more or less a capitalist system. And that’s the only thing you can do to move the system forward” (Healthcare Provider, code 002).*

“*It depends on the facility, personnel, and the quality of service they get that determines the number of patients and even access too, how close or far away the facility is from where people stay. It is the patient voluntarily that determines the facility to choose, not the making of anybody” (Healthcare Provider, code 003).*

### Accreditation of healthcare providers

According to findings from interviewees, it was established that providers interested in joining the scheme will have to apply to obtain the NHIS registration form while the designated accreditation team of NHIS ensures the facility meets the required standard before approval. Below is a quote as reported by the regulator (NHIS):

*“Any healthcare provider that is interested in coming under NHIS will obtain NHIS accreditation form, apply to NHIS, then the accreditation group will be sent to the facility to accredit. There are standards that they must follow. Where we found such been met, then we accredit them. And we accredit for a different group, we have the primary healthcare, secondary and tertiary. A provider may have more than one service; you can have the primary and secondary if all that we needed he could give is found” (National Health Insurance Scheme).*

### Quality assurance

There were various ways of ensuring quality assurance identified by the stakeholders such as establishing the complaint box, dedicated emails, quality assurance unit/department, and forum to discuss as reflected in the quote below:

*“Yes, of course, we have the complaint box there. This place, the NHIS office especially my office have an email address where they can send complaints …*. *We have quality assurance/social health. We have them here. They always go round in the morning to see the patients in the ward, ask them what their complaint is, if they have any complaints, they will jot it down and make sure they address it that day …. We have a forum, am even planning to organize another one by October this year. We did the last one last year October or September. We interact together, play, share feelings, and all that. We here normally do such thing. NHIS do their own, HMO will do their own“(Healthcare Provider, code 001).*

Examples of complaints usually received by stakeholders range from missing names of enrollees at health facilities “*People will come and say they got to their facilities and they said their names are not there …*. O*r they got there; they do not see a qualified doctor to attend to them “(National Health Insurance Scheme) to “there was no drug … …. Also, the issue of not getting services on time If you go to the waiting area, you spend a lot of time“(Healthcare Provider, code 002) to “… delay in giving out authorization code, delay in payment, and facilities collect money from patients that are covered under the scheme” (Healthcare Provider, code 003).*

### Achievement of the scheme

According to the findings from interviewees, opinions were polarised as to the achievement of the scheme among respondents. Some stakeholders acknowledged that the scheme has provided more coverage for the formal sector workers as revealed by the quotes below:

“*NHIS has covered 90% of the formal sector and it has provided affordable and qualitative healthcare to many Nigerians “(National Health Insurance Scheme).*

*“If you do the evaluation of the scheme so far, it has recorded tremendous success for the people that have been covered because a lot of people have been accessing the facility without having money at hand, out-of-pocket expenses” (Healthcare Provider, code 002).*

However, some stakeholders believed that the scheme has performed poorly owing to its poor coverage of Nigerians majorly in the informal sector. The quote below succinctly illustrates this:

“*Health insurance has started in Nigeria but the success is poor if you are rating it. The achievement is like 5% of Nigerians is covered so far. So if you have 5% then you can’t talk about success” (State Health Insurance Agency).*

### Implementation challenges affecting the formal sector

Various challenges affecting the scheme emerged across the stakeholders such as poor enrollee and providers’ knowledge/awareness about the scheme, lack of political will, delay in reimbursement and issuance of authorization code to providers by the HMOs, weak managerial capacity and regulatory oversight functions on the part of the NHIS officials, administrative inefficiency, and voluntary nature of the scheme. The quotes below capture the challenges:

*“… Enrollee enlightenment is very poor, enrollee knowledge about the scheme is very poor, that’s one issue … The hospital themselves, most of them do not understand the scheme, they don’t understand when to request for authorization code, they don’t understand how to prevent the abuse … .. Some HMOs do not give authorization when they are supposed to give authorization and that affects the patient …. Then, the issue of delivering money to the hospital, some HMO has their issues … ..The NHIS themselves are not effective in their monitoring and evaluation of all the other stakeholders, they are not efficient …*. *Then finally, there are loopholes in the law established in NHIS, in a way, the fact that it is not compulsory is limiting spread. Then some people are also going against the law in so many ways especially the NHIS itself. (Health Maintenance Organisation, code 004).*

*“Low level of awareness. The fund is a serious issue, understanding of the concept is another and then getting political will of the government is another serious issue … (National Health Insurance Scheme).*

### Implementation challenges affecting the informal sector

Major challenges as revealed by the stakeholders specifically affecting the informal sector are poverty and non-mandatory nature of the Act establishing the scheme. Many in the informal sector who constitute majority of the population are too poor to enroll into the scheme. Furthermore, the government and the government does not have the authority to make the scheme mandatory as a result of the Act under which the scheme was implemented which has made the scheme a voluntary endeavour.

*“Let me say 70% our populace are suffering from poverty, abject poverty. To do away with little amount is too difficult …. And that is the reason why we find many untimely death among us” (State political leader).*

*“I consider the major lacuna in the national health insurance is that that act did not make health insurance mandatory. So if something is not mandatory, you have given room for failure from the word go” (State Health Insurance Agency).*

Another challenge is that they are not organised coupled with the fact that the mode of payment is not friendly “… *of course again because the informal sector is not well organised, like you can’t say that I want to deduct your premium form source … Also, the mode of payment is not yet friendly … Let me give you an example, a woman that is selling elubo that knows she has a lot of competitors, she doesn’t want to leave her kiosk and say am going to pay in a bank, so she doesn’t want to leave her kiosk otherwise a lot of people that bought” (State Health Insurance Agency).*

Other challenges limiting the buy-in of the informal sector into the scheme include low level of awareness/information about the scheme “… *where the problem lie is that the information is not readily available to the people” (Healthcare Provider, code 003); low interest of the people in the scheme and trust in anything government resulting from personal or religious believes and experiences “…. Then our faith, the Muslim will say they will not do anything insurance not to talk of health insurance. The Christian will say I reject it in Jesus name, I can never be sick … Then the fact that other insurance that has been popular before now is it accident insurance, burglar insurance, fire insurance, has not been faithfully implemented”” (National Health Insurance Scheme).*

### Suggested solutions

As regards the identified challenges hindering the successful implementation of the scheme, stakeholders were asked to suggest solutions and sustainability measures for improving coverage and buy-in of the populace into the scheme. Stakeholders emphasised mass publicity and awareness creation among community members so they can know the benefits of the scheme. Below is a quote to support this.

*“You can go to the communities …. They sensitize them on health insurance, radio, television, jingles and so on are also means of sensitizing people. Even market women, both traditional and modern means of sensitization and publicity can be employed” (National Health Insurance Scheme).*

Also, it was affirmed that there is a need for both legislation to decentralise the scheme to the grass-root level and effective communication synergy between all relevant stakeholders of the scheme. These findings are supported by the quotes below:

*“Stakeholders need to cooperate, among themselves, and they should set a standard of communication skill …*. I *have said it earlier that for NHIS stakeholders to be successful in the scheme they need to have a solid communication strategy” (Healthcare Provider, code 001).*

*“My own perspective of it is that the new step we are taking by legislating a law to make it and break it down to the grass-root it will make to achieve a great success” (State political leader).*

Aside from the solutions identified, stakeholders suggested measures to further sustain the scheme beyond its current level. These include political will from the government, the cooperation of stakeholders, and introduction of co-payment by both government and enrollees to boost funding for the scheme.

“*Well, it’s the political will from the govt. the federal govt. keep on sustaining it and with the cooperation of other key stakeholders like the NHIS, HMOs, and the HCPs. So we need to synergize all of us so that we can keep on sustaining the success story” (Healthcare Provider, 002).*

“*So I will advise that the issue of co-payment should be introduced … The federal Govt. is supposed to be deducting a certain percentage from our salary but as am talking to you they are not” (Healthcare Provider, 003).*

### Reform of the scheme (establishment of state supported health insurance scheme SSHIS)

The reform of the scheme is to bring about state ownership, whereby state governments will establish and run a prepayment scheme for health within each state with the aim of to achieve universal coverage. Stakeholders’ awareness and opinions of the reform, suggestions on how to successfully implement the scheme, strategies for premium collection from the informal sector, and areas of capacity development were assessed.

Few of the stakeholders were not aware of the SSHIS, but notwithstanding, all of them confirmed that they were in support of the reform, and saw it as a good step in the right direction towards achieving the universal health coverage. The quote below shows their views.

*“I have not read about that. But it is not a bad idea provided if they have a good structure on the ground to make sure it’s sustainable. If they have a good structure on ground at least it will widen the scope of coverage of NHIS” (Healthcare provider code 003).*

*“Yes, I am aware of the newly constituted Board of the SSHIS in Oyo State*. *The reform is meant to impact positively towards achieving Universal Health Coverage in Nigeria.”* (*Health Maintenance Organisation, code 006*).

However, stakeholders expressed concerns about the legal framework of the reforms at the state level. It was reported that every state should have a law that makes the scheme compulsory. Additionally, the state should set up an agency to manage the scheme with the support of NHIS. Quote below illustrate their view:

*“Have a draft bill. Ensure that the house of assembly of that state passes the bill, that the bill should mandate the health insurance to all the residents of that state to buy into the health insurance. …. Also, we are encouraging community-based health insurance. It is expected that members of the community will know one another, whom to trust among themselves. … NHIS will equally assist any states that tailor her bill towards the expectation of NHIS. NHIS expects that each state will use TPA- third party administrators to run their health insurance …*. N*HIS will support a bill whereby the board of the state, NHIS will be a voting member …. Then the bill must also encourage the establishment of agency..... Another thing is that any state that NHIS will support is the one that its bill when passed into law will make NHIS mandatory to the residents of the state … ..It’s not been easy to amend our own bill at the federal level to ensure that health insurance is mandatory for Nigerians. And except it is mandatory for Nigerians, you cannot achieve UHC” (National Health Insurance Scheme).*

According to the views expressed by respondents, one major strategy generally reported to ensure the collection of premiums among the informal sector is by encouraging them to form a community/group/association.

“*That’s why we are encouraging community-based health insurance where they pay 1800 naira per year at the rate of 150 naira per month” (National Health Insurance Scheme).*

*“It’s better to have an association because by having an association you start from the landlord, market women, the okada riders, taxi drivers, bricklayers, mechanics, so if we can get through that, at least from their contribution, from their savings, they can use part of their savings or contribution for health care” (Healthcare Provider, Code 001).*

Identified areas of capacity development to ensure successful takeoff of the scheme at the state level include training, ICT, technical and financial assistance. *“It depends on what the state requested. NHIS is available to give any assistance to any state that requested. We have ICT, financial assistance, technical assistance …*.” *(National Health Insurance Scheme).*

## Discussion

In this study, stakeholders’ perspective on the design and implementation challenges of the National Health Insurance Scheme was explored. The study found that NHIS is skewed towards the formal sector raising huge concerns about equity and financial risk protection for those outside the sector. Despite the introduction of Social Health Insurance (SHI) in Nigeria over a decade ago, a greater percentage of health services are paid for through direct user fees [18]. Besides the payment made by voluntary contributors, most federal employees who are covered under the scheme do not co-contribute to the scheme.

This is at variance with payment arrangement between employees and employers as stipulated in the NHIS guideline [15]. This provides federal civil servants with more access to health services than employees in the state and the local government service as well as the self-employed [17]. An exception was made as regards the employees of the National Health Insurance Scheme who have started co-contributing to the scheme in line with the guideline and sustainability model.

Although the majority of the stakeholders believe the capitation is adequate to purchase basic minimum care packages for the enrollees, opposing views came from health care providers who strongly believe that the capitation does not reflect the economic realities of the day hence, it should be reviewed for an increase. The provider-payment system found in this study viz.; capitation and fee-for-service are equivalent to that being practised in Ghana’s NHIS, and although there were complaints by providers on the system according to the study, amount of money was not mentioned unlike that of this study [19]. However, the monthly per capita payment (GH¢1.75 = 130.95 naira) offered to service providers is lower than that found in this study [20, 21]. The provider-payment system should permit the providers to attain a reasonable income, so as to promote good quality service to patients [22].

A key finding was that enrollees are no longer assigned to HMOs as was the usual practice before. Likewise, there is freedom of enrollees to choose their providers as this could have been influenced by the quality of care and services rendered, incentives, location among others. This could lead to lopsidedness with some HCPs having large enrollees base as was found out by a study in Ibadan city where it was observed that more than half of current enrollees are concentrated between just three providers out of the 132 accredited providers [23]. Although stakeholders perceived this to be of advantage in terms of improving competition and quality assurance among the service providers, however, this may equally make other providers with fewer enrollee base feel shortchanged. The importance of health providers as ‘street-level bureaucrats’ whose engagement with patients and the policymaking process can influence policy implementation have been demonstrated in several studies, hence ignoring provider concerns may create an implementation gap especially in the area of consumer quality care [24–26].

There exist a number of measures put in place to ensure the quality of services by the stakeholders. This must have contributed to the high satisfaction of enrollees towards the scheme as reported by [27]. This notwithstanding, other dissatisfying issues especially the delay in processing authorization code by Health Management Organization (HMO) when enrollees are to be referred to secondary or tertiary health facility continue to plague the scheme and negatively impact satisfaction [28].

Previous studies have shown that social health insurance have facilitated access to health care and also have reduced out-of-pocket expenses among members. However, low level of population coverage is common [29–31].

Social health insurance schemes in other sub-Saharan African countries such as Ghana, Senegal and Rwanda have been reported to perform better [9, 32–34]. The poor achievement of the scheme in this regard in Nigeria unlike in Ghana has been attributed to many factors including the type of political institution and structure Nigeria operates [35]. Furthermore, Carrin and James [36] reported the numbers of years of transition it took countries like Austria (79), Belgium (118), Costa Rica (20), Germany (127), Israel (84), Japan (36), Republic of Korea (26) and Luxembourg (72) to reach universal health coverage via social health insurance. However, previous studies have predicted more steady transitions to universal coverage, with coverage levels at 60–80% in just 9 years after implementation [37]. With the present post implementation population coverage of Nigeria at 5% in 15 years, it may take Nigeria about 180 years to implement universal coverage to the same level as those of Ghana, and Rwanda to extend coverage to the self-employed and those on low income levels.

Several challenges serving as major constraints to the successful implementation of the scheme were highlighted making the attainment of universal coverage in Nigeria a far-reaching dream. Some of the challenges are not new and have been reported by previous studies [27, 28, 38–40]. Furthermore, the major lacuna in the Act establishing the NHIS which made it voluntary persists. It creates a loophole for major players and potential enrollees to exploit as compared with what obtains in Ghana, Rwanda, and Tanzania where the SHI is mandatory [9].

Stakeholders’ opinion on how to improve, scale-up and sustainably implement the scheme as found in this study was in line with recommendations of researchers over the years to achieve global best practices with consideration to our local context [27, 28, 38–41]. However, the issue remains how these recommendations are adopted and translated into practice.

According to Cassells [42], actors’ interests and power imbalance among them usually results in conflicts especially when it has to do with resources allocation [43–45]. This was the case of NHIS where most states resisted the adoption of the scheme, attributing it to lack of transparency since they were not allowed in the governance role [46, 47]. Hence the need for reform as this will fast track better implementation of the scheme at both the state and local level thereby reducing the levels of poverty and catastrophic health spending. The findings revealed that not all stakeholders were aware of the reform about the decentralisation of the NHIS to the state level in terms of state supported health insurance scheme (SSHIS). However, the favourable disposition of all stakeholders to the reform is highly commendable, especially considering that reforms involving policies for social health insurance may result in conflicts because the outcome may favour or disfavour various interest groups [37].

Moreover, this reform is similar to what obtains in the following Latin America countries- Argentina, Brazil, Chile, Colombia, Costa Rica, Cuba, Mexico, Peru, Uruguay and Venezuela where health was established as a citizen’s right to expand access to health services, improve health outcome and increase financial risk protection. This is was followed by the introduction of health system reforms with diverse organisational and governance, financing, resource management, and service delivery arrangements [48]. The organisational and governance change led to the reorganisation of health systems, decentralisation of decision making to local levels of government, improvement of regulatory functions, and separation of financing.

The important success factors and strategies for improving coverage among the informal sector as suggested by the stakeholders especially making the scheme mandatory, increasing awareness and encouraging those in the informal sector to form groups/associations and buy-into the community based health insurance may enhance the success of the reform [27, 34, 36, 49]. *“*Although, people may be willing to pay, however, because the majority of them in this environment are poor [9], the capacity to pay the premium, especially on regular basis could be weak [18]. This inadvertently affect a successful and sustainable implementation of the scheme. As there is no data bank of the population outside of the formal sector in Nigeria similar to the situation in the majority of the developing countries especially in the sub-Saharan African countries, this study recommend that efficient platforms to enroll the informal sector be designed. Promoting sub-national schemes such as community based health insurance schemes and the currently approved state supported health insurance program [50] in Nigeria will be of assistance as they are closer to the people than the national scheme (NHIS). The community-based schemes could be re-insured to enhance a more financially viable and stable schemes [51]. Also, subsidies with full fee exemption for the most poor and a sliding –scale premiums [52], for other categories of the poor should be introduced.

Again, poor attitude or low level of interest stems from many factors such as negative superstitious belief about prepayment schemes, [9] and low level of trust in government health interventions [53]. This study recommend genuine efforts to build the trust of the people in government policies. This can be achieved through implementation of beneficial social policies in communities and making efforts not to breach agreements on the part of the government. Negative perception of the people about prepayment schemes could be addressed through intensive and sustained health education and advocacy to the communities of potential beneficiaries [54].

## Conclusion

National Health Insurance Scheme implementation in Nigeria is not effective and efficient. The coverage is skewed and inequitable, especially among the informal sector. Most of the Federal workers do not co-contribute to the scheme in line with the NHIS guideline raising concerns about sustainability and equity. The scheme still faces daunting challenges especially poor awareness about benefit packages among potential beneficiaries, voluntary nature of the scheme, poor service delivery by HCPs, delay in issuance of authorisation code by the HMOs, inefficient mode of payment, and weak administrative capacity of the regulatory body. In addition are wide spread poverty and lack of database for the informal sector, low interest of the people in the scheme, superstitious or religious believes, low adoption rate of reform at the sub-national level, bureaucracy among others.

Amending the NHIS Act, mass sensitisation, capacity building for actors, increase use of ICT, organisation of the informal sector, strict administrative and regulatory oversight have been suggested as ways to improve the effective implementation of the scheme. The various reforms of the scheme at the state level were wholly supported. Hence, there is a need for all stakeholders to work harmoniously to address these challenges. Also, sustained political will is required from policy actors and leaders to back various reforms put in place to attain the milestone of UHC by 2030.

### Limitation

The limited number of key informant interviews (KII) might not allow generalisability of the result. However, with caution, the findings may be useful in similar other settings. This study provides exploratory information on the design and implementation challenges of NHIS in Nigeria.

## Supplementary Information

**Additional file 1.** Key informant interview guide.

## Data Availability

Availability of data and materials may be made available upon reasonable request from the corresponding author. This can only be used for non-commercial purposes which ensures that participants’ confidentiality is protected. Information about study participants will not be made available.

## Abbreviations

NHIS: National Health Insurance Scheme
HMO: Health Maintenance Organization
HCP: Healthcare providers
SSHIS: State Supported Health Insurance Scheme
UHC: Universal Health Coverage
