# Supplementary material for: Assessment of the design and implementation challenges of the National Health Insurance Scheme in Nigeria: a qualitative study among sub-national level actors, healthcare and insurance providers
Source: BMC Public Health. 2021 Jan 11;21:124. doi: 10.1186/s12889-020-10133-5 (PMC7802210; doi:10.1186/s12889-020-10133-5)
Supplement: Supplementary file 1 — Additional file 1. Key informant interview guide. [file 12889_2020_10133_MOESM1_ESM.docx]

KEY INFORMANT INTERVIEW GUIDE

My name is Alawode Gbadegesin Oyewole with the matric number 187615. I am a Masters of Public Health student in the Department of Health Policy and Management, Faculty of Public Health, University of Ibadan. This interview is a process of getting to know the mind of stakeholders like you on the way forward about the government policy and efforts to implement a prepayment scheme for health in Nigeria.

It would be so much appreciated if you could cooperate with me for an estimated period of about 1hour to enable me conduct this interview with you.

1. Yes 2. No
2. Date of Interview ……………………………….
3. Sex of Interviewee ………………………………

If you have any question you are much welcome to do so please.

________________________

Signature

Thank you

I will start the interview now, and will be recorded on a tape recorder to enable me capture all that will be discussed in the course of this interview. Your name or any other identifier that could be used to identify you as a person will not be used in this interview.

Sir, kindly introduce yourself; your position and your organization.

How old are you please?

How long have you worked in this area of the health sector, and, how long have you spent in this particular organization?

In your opinion;

1. What has been the successes achieved by the health insurance in Nigeria so far? And how do you feel it could be maintained?
2. What are the peculiar challenges facing the scheme and, in your opinion, how do you think it could be surmounted?

Probe: What is the present extent of coverage of the formal sector?

What about the informal sector, what are the peculiar challenges and how do you think these challenges could be addressed?

1. What efforts have you been making if any, to reach out to this sector, and what has the response been?
2. In your own opinion, how could the informal sector be made to accept the scheme, and what do you think the stakeholders need to do to achieve and maintain this?

*Specifically ask about* the coverage, fund collection, awareness about the scheme among potential beneficiaries, is it adequate?

1. If the level of awareness is low, what strategies could we adopt to ensure that the level of awareness is raised?
2. What do you think is the attitude of the people in this environment to the scheme and other prepayment schemes for health?

Probe; Are people favourably disposed to it or otherwise,

1. If the attitude is negative, in your opinion, what can we do to address this?
2. In your opinion, what can the stakeholders do to gain the trust of the people in relation to fund management?
3. What about the belief that prepayment scheme is like praying for ill-health, what could be done to make people accept the scheme?
4. Do you think that paying premium can be a challenge among the people?
5. In your own opinion, what could the stakeholders do to solve this challenge?
6. Are there any other challenges that need to be addressed, and how should it (they) be addressed?Distance and the utilization of health facilities in rural Nigeria [^☆^](http://www.sciencedirect.com/science/article/pii/0277953683902988#f)Author links open the overlay panel. Numbers correspond to the affiliation list which can be exposed by using the show more link. [Robert Stock](http://www.sciencedirect.com/science/article/pii/0277953683902988)

Let’s talk briefly about the operations of the scheme in the formal sector as it is presently;

1. What is the process of selecting enrollees under the scheme, do people go and register in organisations of their choice or are names forwarded to organisations?
2. If names are forwarded, by who?
3. What are the pros and cons of these two methods of enrollees’ selection?
4. In your opinion, which method is better?
5. How do you go about selecting Providers, what are the criteria for selecting a particular provider that will render health care to enrollees?
6. How many healthcare Providers do you work with presently within Ibadan City alone?
7. How are the scheme’s enrollees assigned to healthcare providers?

*Probe:* Do they choose healthcare providers or were they assigned to Providers?

1. If enrollees are assigned to providers, what are the criteria for doing this?

*Probe;* What is the current pattern of distribution of enrollees among providers in Ibadan city to be specific, do you know?

(If he does not know, tell him about the fact that > 50% of the current enrollees are shared between just 3providers out of 132 accredited providers)

1. In your opinion, could there be problems associated with this lopsided distribution? Please specify these likely challenges
2. How could the challenges be addressed?
3. How do you reimburse HMOs for the health services purchased for enrollees who registered with them (HMOs)
4. Presently, how much is the premium per capita per month? (*It currently stand at N750 per month per capita*) Does it include administrative charges? If not how much is the administrative charges per capita per month?
5. Who is responsible for the payment, government or the enrollee or both?
6. If it one of the two parties that is paying, why not the other party?
7. In your own opinion, is this amount adequate for a basic health care package or is it inadequate? Please explain why it is adequate (or inadequate).
8. What is the pattern of reimbursement to the HMOs, monthly, quarterly or any other way?
9. How are providers reimbursed?

Probe: Is it for the number of enrollees who accessed services in a particular period or the total number of enrollees who registered with the provider, irrespective of whether they access services in that particular period or not?

1. How do you reimburse the HMOs?

Probe: Is it for the number of enrollees who accessed services in a particular period or the total number of enrollees who registered with the organization, and irrespective of whether they access services in that particular period or not?

1. Is there any avenue through which enrollees can lodge complaints about perceived poor services by the healthcare providers? *Please be specific about this.*

*Probe; Do you have a quality assurance unit/department in your organization? If yes, what are the functions/deliverables of the unit/department?*

1. Have there been complaints from enrollees about perceived poor services rendered by healthcare providers? How have you been handling these? *Please cite example(s)*
2. Is there any forum that provides opportunities for major stakeholders (NHIS, HMOs, and Providers) in this industry to come together and share ideas, challenges and the way forward?

*Probe*: If there is a forum, what are the usual issues that do come up for discussions? How often do such meetings hold?

1. If there is none, what is your opinion about stakeholders having a forum to share ideas? What steps should be taken in establishing one?

Reform at the National Health Insurance Scheme

1. Are you aware about the latest reform in the NHIS? (*If interviewee seems not to be aware, be specific about the recent decentralisation of the NHIS to the States tagged the State Supported Health Insurance Scheme (SSHIS)*.

If the interviewee was not aware of it, ask for his/her opinion about the development and specifically his/her opinion about the likely impact of the reform in the effort to achieve universal health coverage in Nigeria

If the interviewee is aware about it, ask him/her to tell you more about what he/she knows about it (*specifically inquire about the situation in Oyo State whether or not the interviewee is aware about the newly constituted Board of the SSHIS) .*

1. What should the States and other stakeholders do in its design to ensure its success?

*Probe for;*

1. What are your suggestions to ensure an efficient strategy/mechanism in collecting premium from the informal sector populace (because of their peculiar nature of not been on any data bank unlike the formal sector populace)
2. What areas of capacity development would be necessary to build up the states technical team? (Ask about any need for trainings, and organisations that could enhance the capacity of the state team in the successful implementation and sustainability of the scheme
3. In what area(s), in your personal opinion would the NHIS be useful to build and strengthen the state capacity to achieve its mandate on this scheme?

- ^a^Department of Community Medicine, College of Medicine, University of Nigeria, P.O. Box 3295, Enugu Campus, Enugu, Nigeria
- ^b^Department of Sociology/Anthropology, University of Nigeria, Nsukka Campus, Nsukka, Enugu State, Nigeria

Available online 8 December 2009

Thank you for your time. This is the end of the interview.

Distance and the utilization of health facilities in rural Nigeria [^☆^](http://www.sciencedirect.com/science/article/pii/0277953683902988#f)

Author links open the overlay panel. Numbers correspond to the affiliation list which can be exposed by using the show more link.

- [Robert Stock](http://www.sciencedirect.com/science/article/pii/0277953683902988)
